# Nocturnal gastro-oesophageal reflux and respiratory symptoms are increased in sleep apnoea: comparison with the general population

**DOI:** 10.1136/bmjresp-2023-002192

**Published:** 2024-03-25

**Authors:** Össur Ingi Emilsson, Thor Aspelund, Christer Janson, Bryndis Benediktsdottir, Sigurdur Juliusson, Greg Maislin, Allan I Pack, Brendan T Keenan, Thorarinn Gislason

**Affiliations:** 1 Department of Medical Sciences: Respiratory, Allergy and Sleep Research, Uppsala University, Uppsala, Sweden; 2 Faculty of Medicine, University of Iceland School of Health Sciences, Reykjavik, Iceland; 3 Centre for Public Health Sciences, University of Iceland, Reykjavik, Iceland; 4 Department of Sleep, Landspitali – The National University Hospital of Iceland, Reykjavik, Iceland; 5 Department of Otolaryngology, Landspitali—The National University Hospital of Iceland, Reykjavik, Iceland; 6 Department of Medicine, University of Pennsylvania Perelman School of Medicine, Philadelphia, Pennsylvania, USA

**Keywords:** Asthma, Sleep apnoea, Cough/Mechanisms/Pharmacology, Clinical Epidemiology

## Abstract

**Aim:**

To assess respiratory symptoms and nocturnal gastro-oesophageal reflux (nGER) among untreated obstructive sleep apnoea (OSA) patients, compared with the general population. Also, if nGER associates differently with respiratory symptoms among OSA patients.

**Methods:**

2 study cohorts were included: 822 newly diagnosed subjects with moderate–severe OSA and 738 Icelandic general population study participants. All participants answered the same questionnaires. Those reporting nGER symptoms at least once per week were defined as ‘with nGER’; those without nGER symptoms and without nGER medication were defined as ‘no nGER’; and other participants were defined as having ‘possible nGER’. Propensity score-based weights were used to minimise confounding and selection bias and facilitate causal interpretations.

**Results:**

The prevalence of nGER among OSA patients was 14.1%, compared with 5.8% in the general population. This increased prevalence in OSA was not explained by differences in age, gender, body mass index, smoking, hypertension and diabetes (adjusted OR (95% CI)=3.79 (2.24 to 6.43)). OSA patients ‘with nGER’ and with ‘possible nGER’ reported more wheezing (44% and 44% vs 25%, respectively) and productive cough (47% and 42% vs 29%, respectively), compared with OSA patients with ‘no nGER’. The same pattern was seen in the general population, although with a generally lower prevalence. The effect of nGER on respiratory symptoms was similar between the two cohorts.

**Conclusion:**

nGER was more often reported among untreated moderate–severe OSA patients than in the general population. Participants with nGER had more wheezing and productive cough, both among untreated OSA patients and in the general population.

**WHAT IS ALREADY KNOWN ON THIS TOPIC**

- Nocturnal gastro-oesophageal reflux (nGER) and respiratory symptoms are commonly observed among patients with obstructive sleep apnoea (OSA). However, to which extent and how much this is confounded by shared risk factors such as obesity is still unclear.

**WHAT THIS STUDY ADDS**

- nGER and respiratory symptoms are more common among untreated OSA patients than in a comparable general population, independent of shared risk factors. Also, nGER associates independently with more wheezing and productive cough.

**HOW THIS STUDY MIGHT AFFECT RESEARCH, PRACTICE OR POLICY**

- Clinical OSA patients have a higher prevalence of nGER, and OSA together with nGER associates with increased respiratory symptoms. Therefore, having OSA together with nGER may suggest a more burdensome OSA. Further studies on OSA treatment should evaluate OSA-related symptoms such as nGER and respiratory symptoms, in addition to traditional OSA symptoms.

## Introduction

Nocturnal gastro-oesophageal reflux (nGER) and respiratory symptoms are commonly observed among patients with obstructive sleep apnoea (OSA).^1 2^ Both nGER and OSA are strongly related to obesity.^3^ Some have hypothesised that obesity may fully explain the association, but studies addressing this are lacking.^1 2 4 5^ Also, nGER is associated with respiratory symptoms.^1 3 6 7^ One general population study found that among participants with symptoms of OSA, respiratory symptoms were more common if the participants concurrently had nGER^8^; however, no objective sleep measurements were performed to assess OSA.

The potential association between OSA, nGER and respiratory symptoms may be confounded by a number of shared risk factors, such as age, gender and body mass index (BMI).^4 9^ Unfortunately, studies directly comparing nGER and respiratory symptoms between OSA patients and the general population with robust control for confounding factors are lacking. What role nGER has in the association between OSA and respiratory symptoms is therefore still unclear.

The aims of this study were: first, to compare the prevalence of nGER in a clinical moderate–severe OSA cohort to that in an unselected general population of similar age; and second, to compare the association between nGER and respiratory symptoms in these two cohorts, which has not been specifically studied before. We hypothesised that nGER would be more common in a clinical OSA cohort than in the general population. We also hypothesised that the association between nGER and respiratory symptoms would differ between a clinical OSA cohort and the general population, after adjusting for confounding factors.

## Material and methods

Data from two cohort studies were combined for this cross-sectional study, one based on a randomly selected general population sample^10^ and one based on a non-selected clinical OSA cohort,^11^ both based in Iceland. Participants in the clinical OSA study, called the Icelandic Sleep Apnea Cohort (ISAC) study (n=822), completed a home-based sleep study and answered detailed questionnaires including the Basic Nordic Sleep Questionnaire, as previously described.^11–14^ Participants in the general population study, the Burden of Obstructive Lung Diseases (BOLD) initiative (n=738), answered similar questionnaires (the only difference relevant to this study being different wording in the question on asthma diagnosis, see online supplemental material), but did not perform a home-based sleep study.^15^ Further details on these studies are given below and in prior publications.^11–13 15^

10.1136/bmjresp-2023-002192.supp1 Supplementary data

This cross-sectional dataset was used to compare the prevalence of nGER and respiratory symptoms between the two cohorts (ISAC and BOLD), and thereafter to study the association between nGER and respiratory symptoms in both cohorts. Adjustments for confounding factors were performed using propensity score-based weights (see Statistical analyses).

### Patient and public involvement statement

Patients were not involved in the development of study design or recruitment of participants.

### Study cohorts

The OSA cohort comes from the ISAC study, described previously in detail.^11–13 16^ The population consisted of 822 patients diagnosed with moderate-to-severe OSA (Apnea–Hypopnea Index (AHI)≥15 events/hour) in the entire population of Iceland who initiated treatment with positive airway pressure (PAP) from September 2005 to December 2009.^17^ As this was before the general recommendations to register interventional trials beforehand, no such registration was performed for ISAC. A total of 818 participants (99.5%) responded to the questions on gastro-oesophageal reflux, and thereby were included in the current study.

The general population cohort came from the BOLD initiative, a multicentre international study aiming to estimate the burden of chronic obstructive pulmonary disease (COPD) worldwide.^15^ This was a random sample of Icelanders≥40 years old, collected in 2004–2006. The participation rate was 81.2% (762 (53% males) out of 939 invited individuals). Thereof, 738 participants (96.9%) responded to the questions on gastro-oesophageal reflux and were included in the current study. No sleep studies were performed in the BOLD study.

### Nocturnal gastro-oesophageal reflux

The definition of nGER was based on self-reported symptoms and nGER medication use (ATC codes A02BC and A02BA). The following question regarding symptoms in the previous 4 weeks, ‘Do you have heartburn or belching when you have gone to bed?’, was used for defining nGER symptoms.^6 14^ Answers rated on a 5-point scale: never/almost never (1); less than once a week (2); once or two times per week (3); three to five times a week (4); or every day or almost every day of the week (5). Those with symptoms once a week or more often were defined as ‘with nGER’. Those reporting never/almost never (score 1) having nGER symptoms and not using medication for nGER were defined as ‘no nGER’. Those reporting having nGER symptoms less than once a week (score 2) were defined as ‘possible nGER’, as they represent a less well-defined nGER group. Also, those without nGER symptoms but using medication for nGER were defined as ‘possible nGER’, as it was unclear if they had significant nGER with well controlled symptoms or did not have nGER but were using medication for nGER for other reasons (eg, gastritis). This definition is summarised in table 1.

**Table 1:** Overview over classification of nGER groups, by symptoms and reported medications for nGER

	No nGER symptoms	nGER less than once a week	nGER once a week or more often
No nGER medication	No nGER (BOLD: 73% ISAC: 60%)	Possible nGER (BOLD: 8% ISAC: 11%)	With nGER (BOLD: 2% ISAC: 10%)
With nGER medication	Possible nGER (BOLD: 10% ISAC: 13%)	Possible nGER (BOLD: 3% ISAC: 3%)	With nGER (BOLD: 4% ISAC: 4%)

Prevalence in each box given in parentheses for the two cohorts.

nGER, nocturnal gastro-oesophageal reflux.

### Respiratory symptoms and diseases

The questions used for respiratory symptoms have been previously described.^15^ In short, the questions addressed having experienced symptoms such as wheezing or coughing up phlegm (productive cough) in the previous year. Participants reporting productive cough most days for at least 3 months per year, for at least the last 2 years, were defined as having chronic bronchitis.^18^ COPD and asthma were defined based on self-reported diagnosis, symptoms and/or medications (further details on definitions given in the online supplemental file 1).

### Comorbidities

The Epworth Sleepiness Scale (ESS), a brief questionnaire that measures daytime sleepiness, was also assessed.^19^ Participants with ESS Score>10 were considered to have excessive daytime sleepiness.^19^ Smoking history was defined as never, previous or current smoker based on replies to standardised questionnaires.^15^ Hypertension and diabetes were defined based on reported doctor’s diagnosis and current medication. Cardiovascular disease was defined as having or having had myocardial infarction, heart failure or stroke. Height and weight were measured by standardised methods and BMI was calculated as kg/m^2^.

### Sleep recordings in ISAC cohort

Prior to referral for PAP treatment, all patients had a sleep study, as described in previous publications.^11–13 16^ Trained sleep technologists scored all sleep studies at the University of Pennsylvania. Scoring of a hypopnea required a ≥30% decrease in airflow for ≥10 s with ≥4% oxygen desaturation or ≥50% decrease in airflow for ≥10 s with a sudden increase in flow at the end of the event. Scoring of an apnoea required ≥80% decrease in flow for ≥10 s. AHI was calculated as the mean number of apnoeas and hypopneas per hour of recording (excluding upright time). Oxygen Desaturation Index (ODI) was calculated as the number of transient drops in oxygen saturation≥4% per hour of recording. For further details, see previous publications.^11 13^ There were no differences in AHI (p=0.54), ODI (p=0.58), average degree of desaturation (p=0.29) or hypoxia time below 90% (p=0.33) between those with no, possible or definite nGER. Similarly, no differences were seen in AHI or ODI in supine or non-supine position (p>0.80 for all comparisons) (table 2).

**Table 2:** Sleep study results in the Icelandic Sleep Apnea Cohort (clinical obstructive sleep apnoea) cohort by nGER status

Measure	Median (IQR)			P value for overall comparison*
	No nGER	Possible nGER	With nGER	
Apnea–Hypopnea Index (events/hour)	42 (30–59)	41 (30–58)	40 (28–57)	0.54
Oxygen Desaturation Index (events/hour)	30 (22–46)	29 (20–48)	29 (20–42)	0.58
Average desaturation (% desaturation/event)	6.6 (5.8–8.0)	6.6 (5.7–7.7)	6.4 (5.5–7.6)	0.29
Hypoxia Time (min.)	28 (9–65)	27 (10–76)	22 (11–59)	0.33

*P value from ANOVA (analysis of variance) comparing values across the three nGER groups, unadjusted analysis.

nGER, nocturnal gastro-oesophageal reflux.

### Statistical analyses

First, the BOLD and ISAC cohorts were compared regarding overall baseline characteristics and the prevalence of ‘possible nGER’ and ‘with nGER’ in the two cohorts, using t-tests for continuous variables and χ^2^ tests for categorical variables. Also, in the ISAC study, we analysed the association between nGER category and sleep measurement outcomes, using ANOVA (analysis of variance) models (results described above, under subheading ‘Sleep recordings in ISAC cohort’).

Next, we analysed the association between the nGER categories and belonging to the BOLD or ISAC cohort, adjusted for confounders using ‘Covariate Balancing Propensity Score’ (CBPS)-based inverse probability of treatment weights (IPTW) (further described below). We also analysed the association between respiratory symptoms and nGER status separately for the two cohorts, using descriptive statistics and logistic regression models with ‘no nGER’ as the reference group. We then repeated the same logistic regression models adjusted for confounders by using the IPTW approach.

To evaluate whether the relationship between nGER and respiratory symptoms differed in the two cohorts, we performed an interaction analysis in a logistic regression model with respiratory symptoms as the outcome and including nGER group and study cohort as interacting predictors via a product term (*nGER group*×*study cohort*), as well as including main effect terms for each interacting factor. These analyses were adjusted using the CBPS-based IPTW approach. We also performed two sensitivity analyses, one excluding possible outliers (based on IPTW values) and one excluding BMI≥30, as further described in online supplemental file 1.

Acknowledging that the two study cohorts may be intrinsically different, one being a general population cohort and the other a clinical OSA cohort, propensity score methods were used to achieve more balanced comparisons and facilitate causal interpretations. Specifically, we generated a covariate-balancing propensity score relative to the probability of being in the ISAC cohort, based on a model that included a priori defined variables of age, gender, BMI, hypertension, diabetes and smoking history. Using this propensity score, we calculated CBPS-based IPTW for the average treatment effect of the treated (in this case ‘treatment’ refers to belonging to the ISAC cohort). The IPTW method is well established, as long as the propensity score is adequate.^20^ The quality of the propensity score model was assessed by calculating standardised differences, which revealed excellent balance in all included covariates (eg, all standardised differences<0.1; see online supplemental figures 1 and 2).

To evaluate the robustness to unmeasured confounding for observed associations between nGER and respiratory symptoms, we used the recently developed E value approach, which provides an estimate of how strong an unmeasured confounder would need to be associated with both the predictor and outcome, independent of covariate adjustments already performed to fully explain the associations.^21^

A p value<0.05 was considered statistically significant. All statistics were calculated with STATA, V.16.1 for Windows (Stata Corporation, College Station, Texas, USA).

## Results

### Study cohorts

Descriptive comparisons of the two study cohorts are presented in table 3. Participants in the clinical moderate–severe OSA cohort (ISAC) were more likely to be males and had a much higher BMI compared with the general population cohort (BOLD). They reported more comorbidities, and more respiratory symptoms (table 3). Additionally, among participants in the BOLD cohort, those reporting snoring and observed apnoeas had more respiratory symptoms (online supplemental table 1). However, the prevalence of diagnosed COPD and asthma was similar in the two cohorts.

**Table 3:** Baseline characteristics of the general population cohort (BOLD) and OSA patient cohort (ISAC)

	General population cohort (BOLD) (n=738)	Clinical OSA cohort (ISAC) (n=818)	P value
Age (years)	57.1±11.8	54.5±10.6	**<0.001**
Male, %	52.9	81.0	**<0.001**
BMI (kg/m^2^)	27.9±4.9	33.5±5.7	**<0.001**
Smoking history, %			**<0.001**
Never smoker	39.2	27.5	
Previous smoker	42.6	51.4	
Current smoker	18.2	21.1	
Hypertension, %	25.3	45.7	**<0.001**
Diabetes, %	3.0	8.7	**<0.001**
Cardiovascular disease, %	15.3	18.4	0.10
S-CRP, mg/L, median (IQR)	1.27 (0.75–3.25)	2.50 (1.35–4.71)	**<0.001**
S-cholesterol, mmol/L, median (IQR)	5.6 (4.9–6.2)	5.1 (4.3–5.8)	**<0.001**
Epworth Sleepiness Scale	6.0±3.9	11.7±5.1	**<0.001**
Wheeze, %	24.2	32.5	**<0.001**
Productive cough, %	15.7	35.1	**<0.001**
Chronic bronchitis, %	9.2	25.6	**<0.001**
Doctor’s diagnosed COPD, %	4.7	4.8	0.94
Doctor’s diagnosed asthma, %	16.7	16.4	0.86
Current asthma, %	10.9	12.1	0.46
nGER status, %			**<0.001**
No nGER	72.8	59.8	
Possible nGER	21.4	26.2	
With nGER	5.8	14.1	

Values are given as mean±SD for continuous variables and percentages for nominal variables. P values for smoking history and nGER status are χ^2^ comparisons for overall group differences.

Values in bold indicate statistical significance, defined as p-value <0.05.

BMI, body mass index; BOLD, Burden of Obstructive Lung Diseases; COPD, chronic obstructive pulmonary disease; CRP, C reactive protein; ISAC, Icelandic Sleep Apnea Cohort; nGER, nocturnal gastro-oesophageal reflux; OSA, obstructive sleep apnoea; S-, Serum-.

### Comparisons of nGER prevalence

Unadjusted comparisons of nGER groupings between the ISAC and BOLD cohorts are shown in table 3. Participants in the ISAC cohort were more likely to be either ‘possible nGER’ or ‘with nGER’ compared with the BOLD cohort (overall p<0.001). After weighting based on the propensity score, participants in the ISAC cohort were at a non-significant 1.2-fold higher odds of ‘possible nGER’ (OR (95% CI)=1.17 (0.79 to 1.75); p=0.44) and a statistically significant 3.8-fold higher odds of being ‘with nGER’ (OR (95% CI)=3.79 (2.24 to 6.43); p<0.001) compared with the BOLD cohort. These results were similar after excluding participants with very large or very small propensity score weights (see online supplemental table 2). Additionally, among participants in the BOLD cohort, those reporting snoring and observed apnoeas had more often nGER (online supplemental table 1).

### Respiratory symptoms by nGER status

**Figure 1:**
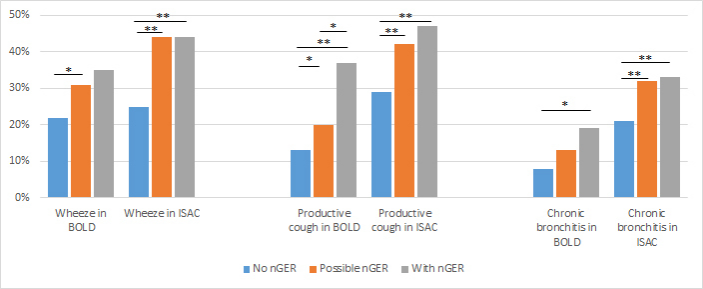
Respiratory symptoms by nGER status in the BOLD (general population) and ISAC (clinical obstructive sleep apnoea) cohorts. (*p<0.05; **p<0.01). BOLD, Burden of Obstructive Lung Diseases; ISAC, Icelandic Sleep Apnea Cohort; nGER, nocturnal gastro-oesophageal reflux.

The prevalences of respiratory symptoms by nGER status, separately by study cohort, are presented in figure 1. In both the BOLD and ISAC cohorts, nGER status was significantly associated with wheezing (p=0.02 and p<0.001, respectively), productive cough (p<0.001 for both cohorts) and chronic bronchitis (p=0.02 and p=0.001, respectively). In general, participants in both cohorts with ‘possible nGER’ or ‘with nGER’ were more likely to report these symptoms than participants with ‘no nGER’ in unadjusted analyses (see figure 1).

To understand whether the relationship between nGER and respiratory symptoms differed in the ISAC and BOLD cohorts, we performed statistical interaction tests with propensity score-based weighting. There was no evidence that the associations between nGER status and the prevalence of wheezing, productive cough and chronic bronchitis differed by cohort (all p≥0.74). The same was true when participants with outlier weights were excluded (online supplemental table 3), and when participants with BMI≥30 were excluded (online supplemental table 4). Being in the ISAC cohort independently associated with a higher likelihood of having productive cough (adj. OR (95% CI): 2.28 (1.15 to 3.60)) and chronic bronchitis (adj. OR (95% CI): 3.55 (2.01 to 6.28)).

**Figure 2:**
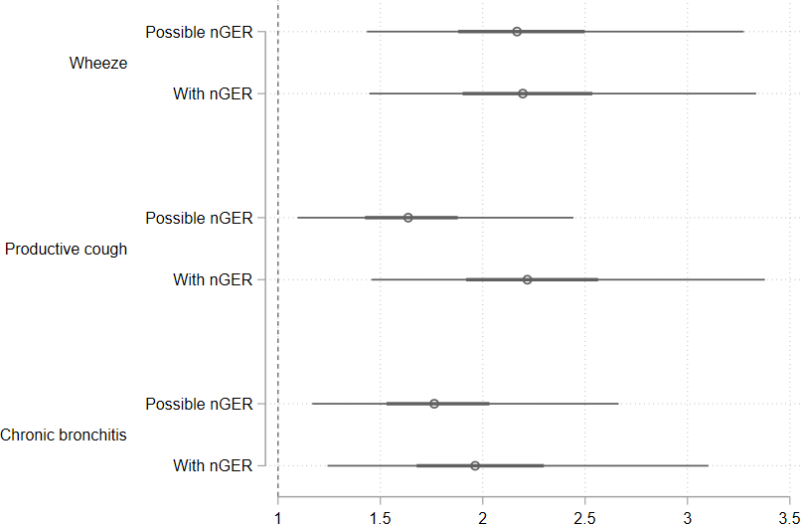
Logistic regression on the association between nGER status and respiratory symptoms, using inverse probability of treatment weighting based on propensity score based on age, gender, body mass index, smoking history, hypertension and diabetes). Results presented as OR with 50% and 95% CI. nGER, nocturnal gastro-oesophageal reflux.

In the two cohorts combined, nGER was significantly associated with wheezing, productive cough and chronic bronchitis, after adjustments using propensity score-based weights (figure 2, online supplemental figures 3–5). Calculation of E value found that these results were moderately robust to unmeasured confounding, as an unmeasured confounder would need to have OR of at least 1.9–2.3 with both the exposure and outcome, independent of included covariates, to fully explain these results.

## Discussion

In this study, we found that untreated moderate–severe OSA patients reported nGER nearly 2.5 times more often than the general population, independent of differences in age, gender, BMI, hypertension, diabetes and smoking history. Also, OSA patients had more wheezing, productive cough and chronic bronchitis than the general population. Among OSA patients, those with nGER reported more wheezing, productive cough and chronic bronchitis, compared with OSA patients with no nGER. Similar associations between nGER and respiratory symptoms were seen in the general population. These associations were not explained by inherent cohort differences, as evidenced by consistent associations in a propensity score-based IPTW analysis. Additionally, the fact that any unmeasured confounder would have to have an association to both nGER and respiratory symptoms with an OR of at least 2.0 (or, equivalently, 0.5) independent of the included confounders seems unlikely. For example, the association of possible unmeasured confounders such as alcohol abuse and physical activity have in other publications had a weaker association, with ORs between 0.70 and 0.98 (or, equivalently, 1.02 and 1.43).^22 23^

### Prevalence of nGER among untreated OSA patients compared with the general population

We found the prevalence of nGER to be 5.8% in the general population cohort, compared with markedly higher nGER prevalence of 14.1% in the unselected clinical moderate–severe OSA cohort. This clear difference was still evident after robustly adjusting for potential confounders (using a propensity score-based IPTW analysis). As we did not have sleep studies in the general population cohort and OSA is relatively common in the general population,^24^ we assume that some general population participants had OSA. Therefore, the difference found reflects how clinical patients with OSA differ from an unselected general population, rather than a general population without OSA (where the difference is likely even larger). Regardless, our data support the conclusion that nGER is more common among untreated moderate–severe OSA patients than in the general population.

Interestingly, even though nGER was more common in our moderate–severe OSA cohort, we did not find associations between nGER and common sleep parameters such as AHI (table 2). This suggests factors other than the number of apneic/hypopneic events may cause the increased nGER among patients with moderate-to-severe OSA. Also, the possibility that the association between nGER and OSA may be bidirectional may influence this result.^25^ Other possible mechanisms have been postulated, such as increased respiratory effort during sleep, which strains the lower oesophageal sphincter muscle.^3 8^ In turn, a weaker lower oesophageal sphincter may cause increased nGER.^26 27^ Therefore, nGER could be a consequence of moderate–severe OSA with more pronounced nocturnal respiratory effort, but studies addressing this are lacking. The current gold standard for measuring respiratory effort is by oesophageal pressure, but this is uncomfortable and rather invasive.^28^ Better screening and measuring methods are therefore needed to evaluate respiratory effort. Clinical signs may also be helpful to indicate which patients with OSA may have significantly increased respiratory effort. Our results together with above-mentioned studies suggest that nGER may be one such indicator.

### Respiratory symptoms and nGER

We found that nGER was associated with a higher prevalence of respiratory symptoms such as wheezing and productive cough, both among untreated OSA patients and in the general population. Having clinically significant OSA did not alter the associations between nGER and respiratory symptoms compared with the general population, but both nGER and respiratory symptoms were more common among the OSA patients. This may indicate that OSA unmasks a predisposition for nGER, which in turn impacts respiratory symptoms.

Regarding nGER, our previous studies have found an association between nGER and respiratory symptoms, especially among snorers and found persistent nGER to be a risk factor for developing respiratory symptoms.^1 6 29^ There are different theories on how nGER may cause respiratory symptoms, and different mechanisms may associate with different respiratory symptoms.^8^ Nocturnal reflux episodes are often long-lasting, and reach further up the oesophagus,^30 31^ which in the context of OSA may increase the risk of microaspirations into the lungs, but this theory has not been studied.

Collectively, we hypothesise that moderate–severe OSA may cause respiratory symptoms, either directly or mediated through nGER secondary to OSA. Further studies are needed to evaluate how OSA treatment may affect the association between nGER and respiratory symptoms.

### Strengths and weaknesses

The main strength of this study lies in the large and well-characterised, unselected, clinical moderate–severe OSA cohort, with a demographically similar general population cohort as a reference group, making the results broadly generalisable. Additionally, a similar trend was found in the general population when stratified by the presence or absence of OSA symptoms (snoring and/or apnoeas). Also, applying propensity score-based methods to adjust for inherent cohort differences and using the E value to demonstrate relative robustness to unmeasured confounding further strengthens the validity of the results.

However, a few methodological weaknesses need to be raised. First, we had no objective measurement for nGER. The questionnaire-based definition used has, however, been used in numerous previous studies and performs reasonably well to identify significant nGER.^1 32 33^ Second, the question on asthma diagnosis was notably more inclusive of asthma-related conditions in the general population cohort compared with the OSA cohort, limiting the possibility for detailed analysis of asthma prevalence between cohorts. Third, we did not have information on other possible confounding factors such as alcohol abuse and physical activity. However, for such an unmeasured confounder to fully explain away the associations found between airway symptoms and nGER beyond the measured confounders, its association to both the predictor and outcome would have to be at least an OR of 1.9–2.3, which seems unlikely for potential unmeasured confounders given previous literature. Also, individuals with OSA could not be excluded from the general population cohort as we lacked sleep studies or information on previous OSA diagnosis in that cohort, which likely lead to weaker associations than if only a non-OSA general population had been compared. The fact that we nonetheless found significant associations strengthens the conclusions.

### Conclusion

We found that nGER was nearly 2.5 times more common among moderate–severe OSA patients than in a similarly aged general population cohort, a result not explained by differences in key covariates. Respiratory symptoms were increased in prevalence among OSA patients, and especially among those with OSA and nGER. Interventional studies are needed to answer how treatment affects the combination of OSA, nGER and respiratory symptoms.

10.1136/bmjresp-2023-002192.supp2 Supplementary data

## Data Availability

Data are available upon reasonable request.
