# Supplementary material for: Nocturnal gastro-oesophageal reflux and respiratory symptoms are increased in sleep apnoea: comparison with the general population
Source: BMJ Open Respir Res. 2024 Mar 25;11(1):e002192. doi: 10.1136/bmjresp-2023-002192 (PMC10966810; doi:10.1136/bmjresp-2023-002192)
Supplement: Supplementary data [file bmjresp-2023-002192supp001.pdf]

ONLINE SUPPLEMENT

Nocturnal Gastroesophageal Reflux and Respiratory Symptoms are increased in Sleep Apnea

- Comparison with the general population

SUPPLEMENTAL METHODS

Definition of COPD and asthma

Participants were also asked if they had been diagnosed with chronic obstructive pulmonary disease (COPD) or asthma. The question on asthma diagnosis differed somewhat between the two cohorts; the ISAC question was "Have you been diagnosed with asthma?" and the BOLD question was "Has a doctor ever said you have asthma, asthmatic bronchitis, or bronchitis because of allergy?". A positive reply to these questions in each cohort was defined as having doctor-diagnosed asthma. Participants with doctor-diagnosed asthma, who additionally reported wheezing and/or current use of asthma medication (ATC code R03) were defined as having current asthma. Consequently, participants who reported doctor-diagnosed asthma, but without wheezing and current use of asthma medications, were defined as not having current asthma.

Sensitivity analyses

Outlier weights for sensitivity IPTW analysis were defined as follows: In total, 1,527 individuals had weights calculated. Weights were first standardized to 1. Natural log transformation of the weights gave a normal distribution of the weights (**Figure S2**). Outliers were defined as having natural log transformed weight value above 2 or below -2 (in total 159, all from the general population BOLD cohort), leaving 1,368 individuals for the sensitivity analysis.

Secondly, sensitivity analysis was also performed where participants with BMI≥30 were excluded.

Subgroup analysis by sleep symptoms in the general population cohort (BOLD)

A portion of the participants in the general population BOLD cohort (n=505, 65%) answered at least one of two questions regarding sleep symptoms: snoring and observed apneas. Snoring was considered present if reported present three nights per week or more. Apneas were considered present if reported present one night per week or more.

SUPPLEMENTAL TABLES

Table S1: Respiratory and nGER symptoms in the general population cohort (BOLD), by reported sleep symptoms (snoring and/or observed apneas)

	**No nocturnal symptoms (n=346)**	**Single OSA symptom (n=128)**	**Two OSA symptoms (n=31)**	**P-value**
Wheeze, %	20.8	31.3	38.7	**0.01**
Productive cough, %	14.6	11.7	35.5	**0.004**
Chronic bronchitis, %	7.6	7.0	29.0	**<0.001**
nGER status, %				**<0.001**
No nGER	79.0	63.8	41.9	
Possible nGER	16.4	28.4	45.2	
With nGER	4.7	7.9	12.9	

Table S2. Association between nGER status and study cohort, analyzed by separate logistic regressions with No nGER as reference group, weighted based on a principal component score and for measuring average treatment effect of the treated. Participants with outlier weights excluded. Results presented as OR (95%CI).

	**General population cohort (BOLD) (n=558)**	**Clinical OSA cohort (ISAC) (n=810)**
Possible nGER	Ref	1.08 (0.78 – 1.50)
With nGER	Ref	2.87 (1.72 – 4.78)

Table S3. Logistic regression on the association between nGER status, cohort and respiratory symptoms, using inverse probability of treatment weighting*. Participants with outlier weights excluded (n=159).

**Symptom**	**P-value for interaction****	**Odds Ratio (95% CI)** †		
		**Possible nGER**‡	**With nGER**‡	**ISAC (OSA) cohort**§
Wheeze	0.56	**1.99 (1.43, 2.76)**	**2.16 (1.45, 3.24)**	0.94 (0.69, 1.28)
Productive cough	0.69	**2.04 (1.45, 2.87)**	**2.43 (1.62, 3.64)**	**2.52 (1.74, 3.64)**
Chronic bronchitis	0.71	**1.98 (1.40, 2.81)**	**2.04 (1.31, 3.16)**	**3.64 (2.43, 5.47)**

*IPTW using propensity score based on age, gender, BMI, smoking history, hypertension, and diabetes; **p-value from interaction between nGER group and cohort, testing whether the association of nGER with symptom measure differs between ISAC and BOLD; †Odds ratio from model including main effect from nGER and cohort, without interaction term; ‡Compared to no nGER; §Compared to BOLD.

Table S4. Logistic regression on the association between nGER status, cohort and respiratory symptoms, using inverse probability of treatment weighting*. Participants with BMI≥30 excluded (n=768, thereof 570 from the ISAC cohort).

Symptom	P-value for interaction**	Odds Ratio (95% CI) †		
		Possible nGER‡	With nGER‡	ISAC (OSA) cohort§
Wheeze	0.31	**2.27 (1.44, 3.60)**	1.51 (0.69, 3.29)	1.29 (0.85, 1.95)
Productive cough	0.33	**2.28 (1.41, 3.70)**	2.05 (0.93, 4.49)	**2.12 (1.37, 3.29)**
Chronic bronchitis	0.69	**2.87 (1.69, 4.88)**	1.71 (0.68, 4.30)	**2.51 (1.51, 4.16)**

*IPTW using propensity score based on age, gender, BMI, smoking history, hypertension, and diabetes; **p-value from interaction between nGER group and cohort, testing whether the association of nGER with symptom measure differs between ISAC and BOLD; †Odds ratio from model including main effect from nGER and cohort, without interaction term; ‡Compared to no nGER; §Compared to BOLD.

SUPPLEMENTAL FIGURES

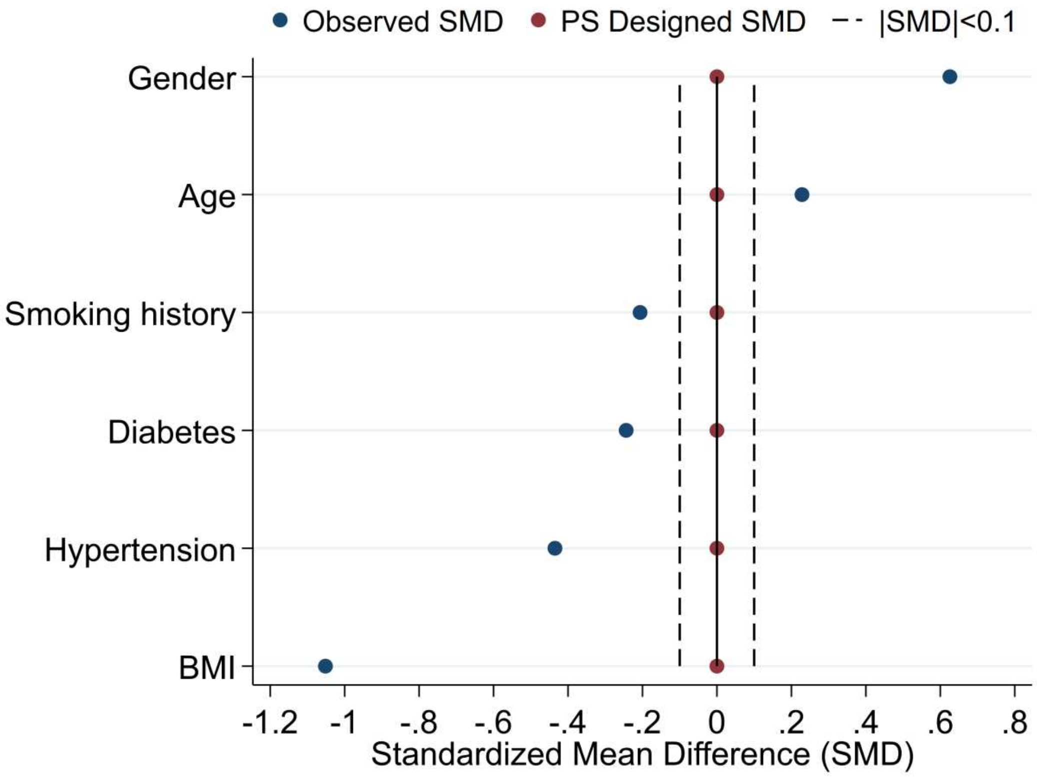

1. Love plot for standardized mean differences of variables included in the propensity score, before and after propensity score weighting. PS: Propensity score; BMI: Body mass index.

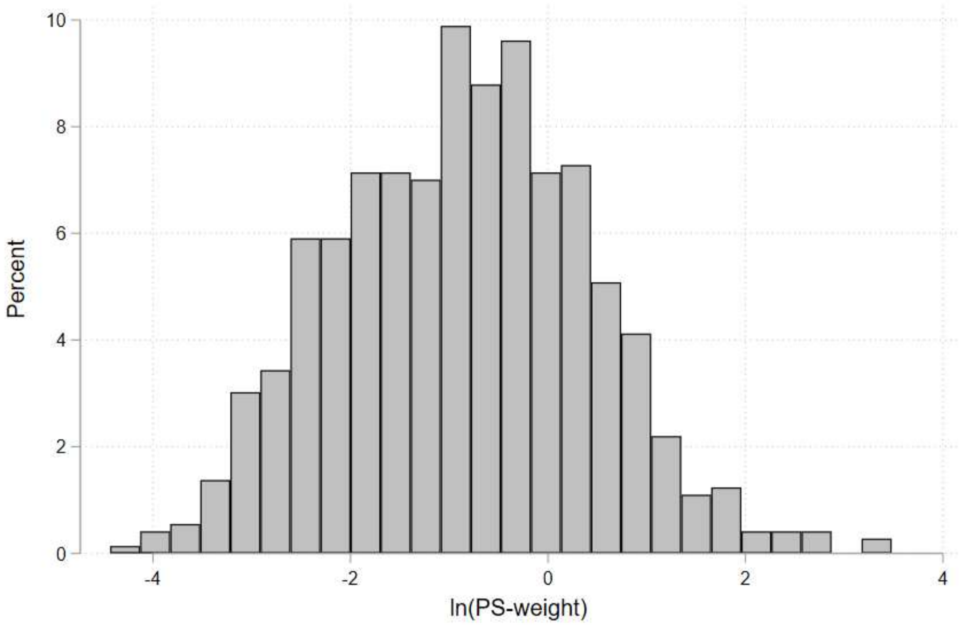

Figure S2. Histogram for ln-transformed weights in the BOLD cohort (all participants in the ISAC cohort had ln(PS-weight) set to 0, as defined by the 'average treatment effect of the treated' method).

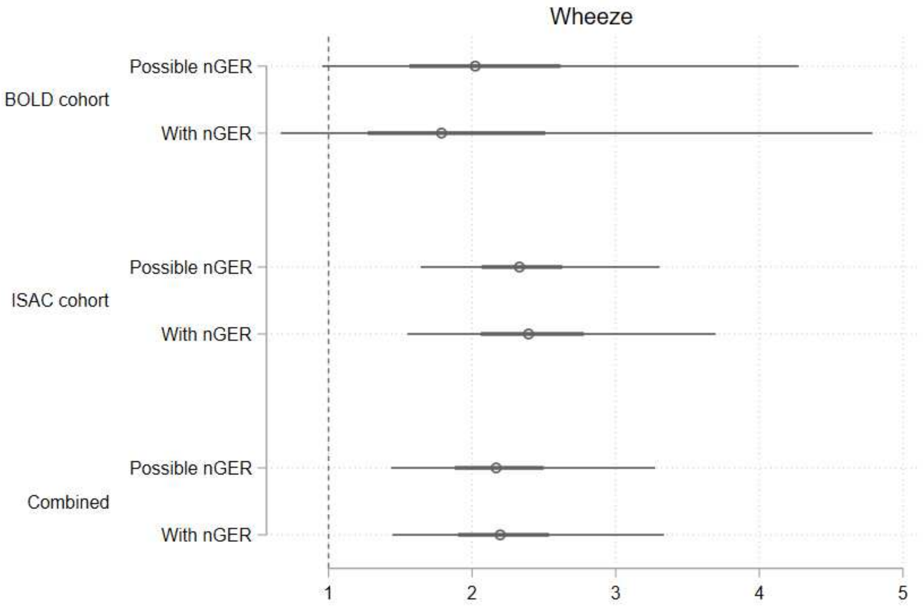

Figure S3. Logistic regression on the association between nGER status and wheeze, using inverse probability of treatment weighting (IPTW), based on propensity score based on age, gender, BMI, smoking history, hypertension, and diabetes. Results presented as Odds Ratio with 50% and 95% CI, for study cohorts separately and combined.

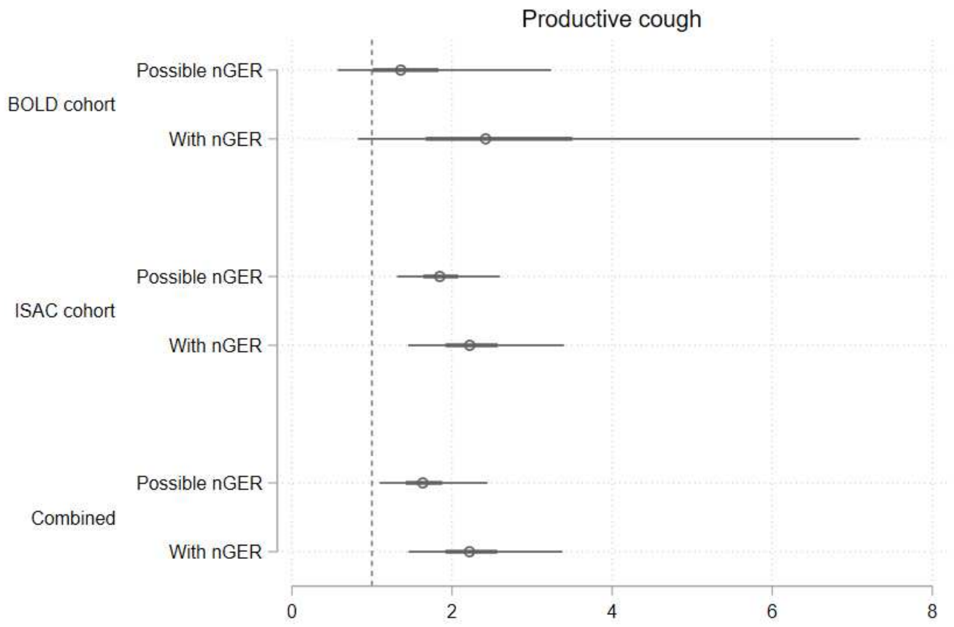

Figure S4. Logistic regression on the association between nGER status and productive cough, using inverse probability of treatment weighting (IPTW), based on propensity score based on age, gender, BMI, smoking history, hypertension, and diabetes. Results presented as Odds Ratio with 50% and 95% CI, for study cohorts separately and combined.

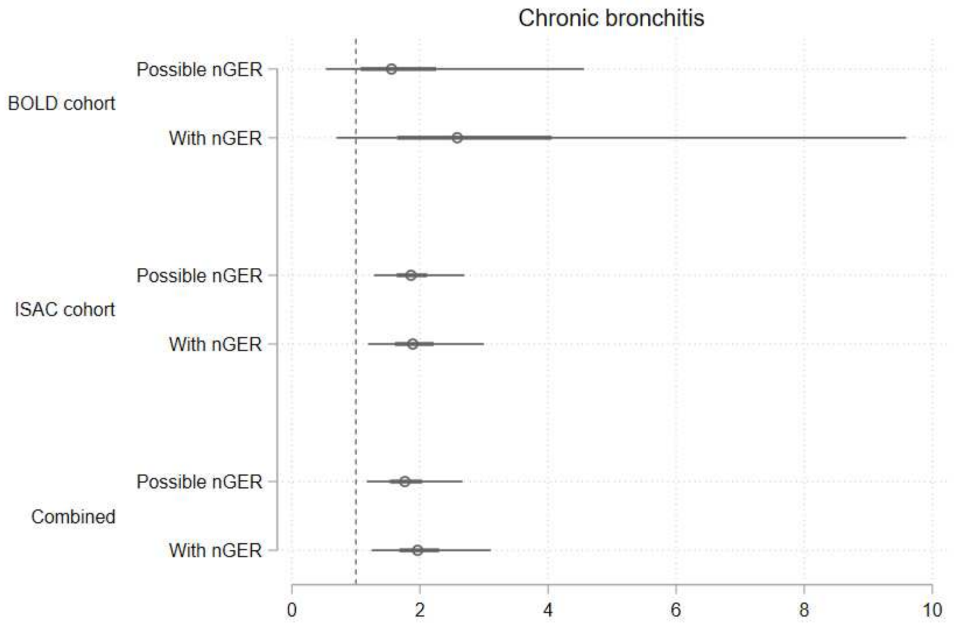

Figure S5. Logistic regression on the association between nGER status and chronic bronchitis, using inverse probability of treatment weighting (IPTW), based on propensity score based on age, gender, BMI, smoking history, hypertension, and diabetes. Results presented as Odds Ratio with 50% and 95% CI, for study cohorts separately and combined.
